# Supporting complex mental health care and services research today and beyond: a mini-review of real-world evidence strategies and informed approaches

**DOI:** 10.3389/frhs.2026.1777597

**Published:** 2026-03-24

**Authors:** Rachele M. Hendricks-Sturrup, Fraser W. Gaspar

**Affiliations:** 1 Duke-Robert J. Margolis, MD, Institute for Health Policy, Washington, DC, United States; 2 The MITRE Corporation, McLean, VA, United States

**Keywords:** complex care, mental health, psychiatry, real-world data, real-world evidence

## Abstract

Global mental health needs are escalating, yet few people with mental disorders receive effective care, underscoring the need for robust real-world evidence (RWE) to guide system transformation. Real-world data (RWD)—such as electronic records, claims, patient-reported outcomes, and digital sources—can capture the complexity of mental health care delivery beyond trials but remain underused. In this mini-review, we discuss methodological and infrastructural priorities for leveraging RWD to improve mental health services research and care. We describe recent peer-reviewed studies that have used RWD to examine complex mental health care and associated outcomes, focusing on applications of artificial intelligence and machine learning (AI/ML) and on approaches that enhance validity and reproducibility. Many recent studies report the use of AI/ML to identify study populations, extract unstructured clinical information, or predict treatment patterns, while others report the use of RWD to characterize trajectories, service use, and costs. Building on these examples, we propose two urgent actions: (1) adopt relevant, reliable, and routine RWD curation, transformation, and analysis strategies—including target trial emulation for causal inference—and (2) strengthen mental health care data systems through standardization, harmonization, and interoperability. To promote transparency, we highlight protocol and reporting tools (e.g., HARPER, ATRAcTR, TARGET) and recommend registration of RWE studies. Collectively, these advances can enable high-quality, patient-centered RWE that better reflects real-world mental health care and informs more equitable, effective services.

## Introduction

Globally, 332 million cases of mental disorders were estimated in 2021, and depression and anxiety disorders were responsible for 6.2% and 4.7% of all-cause years lived with disability, respectively (1). In September 2025, the World Health Organization (WHO) data showed that over one billion people are living with mental health disorders, prompting the organization to call on “governments and global partners to urgently intensify efforts toward systemic transformation of mental health care systems worldwide(2). The WHO's call for global change is warranted, as overall only 7% of individuals with mental disorders receive effective treatment, indicating significant gaps in care globally even for those with potential confirmed diagnoses (3). Policy and regulatory initiatives, such as the United States (US) 21st Century Cures Act of 2016 and the Cures 2.0 Act draft (released in June 2021 and referred to the US House of Representatives in November 2021), prioritize improving and supporting mental health care services (including substance use disorders) and research, in part through increased use of real-world evidence (RWE) (4, 5). Since then, RWE generation remains a viable, publicly-privately incentivized, and highly prioritized pathway to identify and address significant gaps in mental health care and services research.

RWE is generated from real-world data (RWD), defined as data collected outside controlled clinical trial settings that reflect everyday patient or usual care (6). Data sources for RWD include, but are not limited to, the following:

- Electronic health or medical records
- Payer claims
- Patient-reported outcomes or measures
- Direct-to-consumer testing
- Mobile devices and wearables
- Social media
- Registries
- Laboratory testing
- Pharmacy claims

Given the broad nature of RWD, it can often reflect the realities around mental health care service provision, or lack thereof, within and across geographies. Yet RWD remains underutilized as a resource that can provide real-world context to confirm or refute clinical assumptions and thereby enable more precise action and intervention along the patient journey. Utilizing RWD more meaningfully in mental health services research should therefore be an essential first step in managing and addressing the growing burden of mental illness at the population level.

In this mini-review, we provide a general background on the complexity of mental health services research and care and the challenges/opportunities for using RWD in research. We highlight recent work presenting RWD and RWE study methodologies that are capable of addressing these complexities. Finally, we describe strategies to advance generalizable and actionable RWE, holistic mental health care, and robust mental health services research.

## Complexity in studying mental health services and care

Mental health care often involves complex psychiatric and other individualized or specialized treatment pathways that require robust, long-term, and accessible social support and clinical care. This includes community-level and caregiver support for daily living (including education and employment), specialized clinical care, and routine primary care, especially for patients with significant mental impairments or health risks. However, these systems can be fragmented and resource-constrained, or even nonexistent for some patients. Adding to this complexity are health system and geographic level variation in mental health care standards, creating more challenges for attempts to systematically study and act on study findings reflecting diagnosis patterns, treatment initiation and adherence, symptom severity, functional outcomes, and long-term impacts (7). Inconsistent tracking of these outcomes outside of randomized controlled trials (RCTs) also presents challenges for evaluating treatment effectiveness in real-world settings and for supporting the translation or replication of clinical trial results from controlled settings into routine care (8, 9).

Given the complexity of mental health services research, one viewpoint of RWD is that the underlying data is useful for hypothesis-generation, but not sufficient to accurately answer important mental health research questions including treatment effectiveness. Individuals who hold this viewpoint may believe that RWE can never sufficiently control for confounding, and evidence should primarily come from RCTs. However, there is a vast and growing amount of digitized or machine-readable RWD reflecting mental health status and complex care patterns. To accommodate this form of technological growth and accurately capture this complexity, sound research methodologies and epidemiology research tools are necessary. These tools can help health systems understand the real-world effectiveness of a variety of standard of care and advanced treatment modalities, alone or combined, and help health systems take evidence-based action. For example, mental health symptom severity has historically been documented in unstructured data, presenting an analytical challenge to extract that clinical information to control in statistical models. More recently, large language models have also shown promise in identifying symptoms captured via unstructured clinical record text (10), and therefore should be further developed and utilized to abstract RWD on mental health symptoms and care.

RWD reflecting patient access to more advanced forms of treatment, like next-generation pharmacotherapies, pharmacogenomic testing, immunomodulation therapies, and electroconvulsive therapy, can be sparse, fragmented, or absent. This is inherently due to the rare or fragmented occurrence of patient access to those forms of treatment (7). Despite this challenge, RWD has the ability to pool data across multiple geographic locations and target populations to perform observational studies of treatment effectiveness when treatment is rare (11). Another challenge for studying mental health care is that treatment, in general, requires months to years of sometimes harmful initial step-therapy processes to both rule out standard treatment-resistant mental illness or treatment interactions, and thus substantiate the need for more advanced treatment options (12–14). The longitudinal nature of RWD, beyond the traditional timeframes used in RCTs, does allow for researchers to assess previous treatment courses when assessing the treatment of interest.

Lastly, variation in interoperability, plus semantic and syntactic elements and features, across electronic health data capture systems can create and perpetuate challenges (15). This ultimately affects researchers' and clinicians' abilities to extract, abstract, curate, harmonize, and transform RWD into actionable RWE for target end-users (15). To minimize the bias from variation in interoperability, RWD researchers should perform appropriate data quality checks including using applicable frameworks such as the Kahn and Patient Information Quality Improvement frameworks (16, 17).

**Figure 1:**
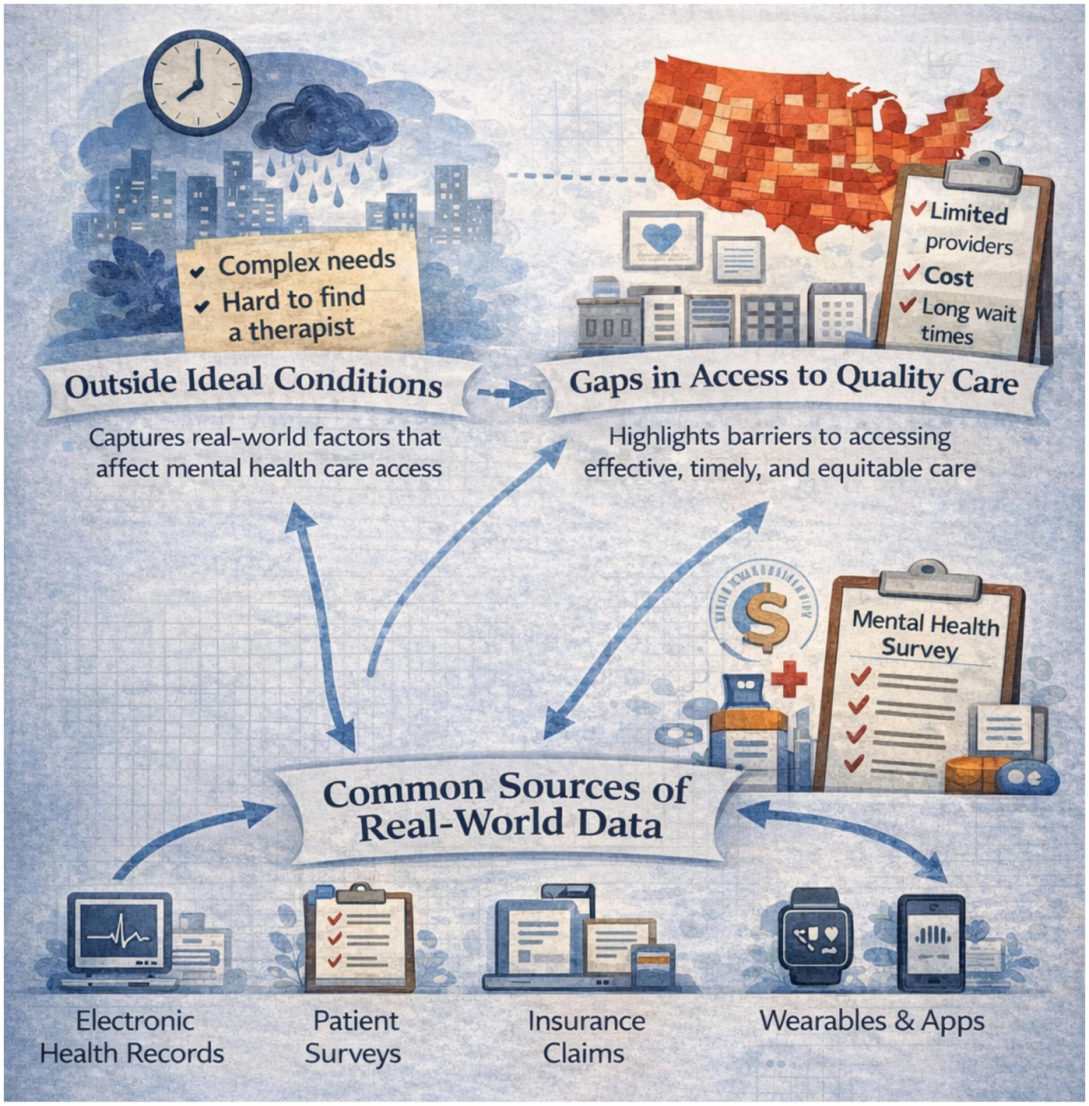
Importance of real-world data in mental health services research and care.

Taken together, although mental health research is complex, RWD can support a better understanding of mental health care delivery complementary to controlled research settings. Figure 1 illustrates how common RWD sources can be used to generate RWE that characterizes care under non-ideal conditions (e.g., complex needs and system constraints), identify gaps in access to quality care, and capture heterogeneity across diverse populations and lived experiences. Arrows within the Figure indicate the flow of information from RWD sources to support or inform key domains of scientific inquiry. The domains include understanding complex health needs, accounting for difficulties patients face in finding mental health therapists, and identifying structural or geographic gaps in access to quality care.

Two key actions are therefore needed today to help promote broader reproducibility, translation, and acceptance of RWE and enhance mental health services research validity:

1. Develop or specify relevant, reliable, and routine mental health RWD collection curation, transformation, and analysis approaches and protocols; and
2. Identify, create, or improve mental health-relevant data systems, including data system interoperability features, to capture the full range and scope of mental health care.

Through these actions, research can drive better mental health care and service by setting up studies to more accurately reflect real-world phenomena. Below, we discuss current and potential future research developments in the field of leveraging RWD and RWE study methodology, with or without the use of AI/ML. We center this discussion around a longstanding and growing demand and need to capture the complexity of mental health care and inform meaningful plans for action. Lastly, we revisit the key actions to describe RWE study methods, tools, resources, and pathways that can help researchers take our proposed actions and collaborate with patients and clinicians to measure burden complexity and achieve desired clinical outcomes. We present this work with the intent to inform and inspire future work along with our special editorial issue focused on advancing mental health research and care using real world data (18).

## Current advances in real-world evidence study methods to assess complexity in mental health care

In Table 1, we highlight a few RWE studies that have sought to capture complexity in mental health care and treatment and report clinical outcomes and/or considerations for innovative study methods. Many of these studies report the use of AI/ML applications to process and evaluate RWD from registries (19), wearables (20), insurance claims (21–23), prescription records (prescribing and dispensing) (19, 24), EHRs (structured and unstructured) (22, 25, 26), and clinical notes (25, 27). RWD sources were evaluated for a multitude of study goals and purposes, including evaluating a retrospective, observational dataset to develop a bipolar disorder patient journey clustering technique and novel cluster quality metric reflective of real-world patient journeys (21) and exploring how common clinical session summaries are in real-world behavioral treatments (27).

**Table 1:** Real-world evidence studies capturing and measuring complexity in mental health care and treatment, and reporting clinical outcomes and/or considerations for innovative study methods.

Author (year of publication)	Burden complexity measured/addressed (RWD source)	Key study outcomes (limitations)	AI/ML use
van Schothorst et al. (2025) (28)	Reflected on lessons learned from a Netherlands study assessing the effectiveness and implementation of a multidisciplinary, multicomponent lifestyle-focused intervention (MULTI+) for inpatients with mental illness. MULTI+ involves 10 core components adaptable to specific contexts and multidisciplinary staff (Electronic patient records derived from a pharmacy system).	Recommendation to standardize data entry procedures by using short, harmonized screening tools; aligning data collection with routine care pathways; and using automated checks to monitor data completeness in real time, thereby prompting corrective action during vs. after study completion [High degree of time, effort, and human resources required to address fragmented and incomplete data (missing on average at 44%), resulting in a discontinuation of this process; Substantial contextual and technical expertise required to access, extract, and harmonize RWD].	None.
Pérez et al. (2025) (19)	Used target trial methods to estimate the long-term causal effect of initiating ADHD medication on national test scores in the domains of English, numeracy, and reading in Norwegian children diagnosed with ADHD. A rich set of covariates and negative control exposure analyses were used to minimize confounding. Long-term “as-started” effect was the chosen estimand to inform general policy effectiveness [Registry data derived from multiple Norwegian-specific sources (*n* = 8,458)].	On average, medication initiation had a small positive effect on standardized national test scores (grades 5 and 8) in the domains of numeracy, reading, and English. Minimal effect was observed across test domains, though numeracy showed a slightly more positive effect. Chosen estimand, however, does not address whether ADHD medication adherence improves academic achievement (Causal interpretation assumed no unobserved confounding).	An algorithm was used to estimate daily dosages and treatment duration, thereby introducing uncertainty.
Alkurdi et al. (2025) (20)	Quantitatively assessed whether and how ML models for anxiety detection built using wearables data in controlled conditions could be applied to real-world environments (U.S. study). Also assessed whether data types influence the effectiveness of the models (Wearables data derived from a wristband and smart shirt).	The traditional learning model was a feature based model, given that prior work has shown that the most robust machine learning models in predicting anxiety from a noise-augmented dataset apply feature based models. Assessment showed the XG Boost model achieved a 0.99 accuracy and *F*_1_ score in the WESAD dataset, whereas performance was maintained across the RADWear and WEAR calibration subsets. Transfer learning model assessment showed that best performance was provided by a RF anxiety classifier, with an *F*_1_ score and accuracy ranging from 0.76 to 0.94 for all datasets (Selected datasets might not fully capture the entire spectrum of individual variability in anxiety responses. Further model validation is needed to determine long-term reliability and usefulness in clinical practice).	Traditional ML and transfer learning models trained to become resilient to real-world, environmental disturbances.
Littman et al. (2025) (21)	Conducted a retrospective, observational study involving a large patient sample within a U.S.-based commercial database. Described the development of a patient journey clustering technique uniquely adapted to psychiatric information, focusing on bipolar disorder, to support future investigations and develop a novel cluster quality metric reflective of real-world patient journeys (Claims data (*n* = 31,578)]	Variability in line of therapy, demographics, comorbidities, and mental health–related HRU and costs were observed across treatment journey clusters. The majority of Cluster 3 patients were treated with mood stabilizer lamotrigine upon initial depressive episode (aligned with clinical recommendations) and did not experience another episode on average during follow-up. Cluster 13 patients did not receive pharmacologic treatment between index manic episode and subsequent depressive episode (not aligned with clinical recommendations); timing between the mania and depression events required further investigation. Costs, average number of bipolar events, mental health-related inpatient and outpatient HRU were substantially higher in Cluster 13 compared to Cluster 3 (Claims data may not have captured all important events along the patient journey. Analysis design and use of claims data may have introduced selection bias—in one sample, patients remained insured throughout our study, which may not be generalizable to all real-world psychiatry populations).	Designed an ML algorithm (kernel *k*-means approach) to accommodate sequences of uneven length with a variety of categorical clinical events.
Hang Lo et al. (2025) (24)	Defined a phenotype of switching from an SSRI to another antidepressant (of any class; ≤90-day gap between prescriptions for an SSRI and another antidepressant in primary care) during a single episode of depression. Characterized prescription patterns and investigated clinical, demographic, and polygenic predictors of SSRI switching [Prescribing record data in the UK Biobank (*n* = 38,813) and dispensing record data in Generation Scotland (*n* = 1,777) study database].	Most SSRI switches occurred within 6 weeks of index prescription in both the UK Biobank and Generation Scotland cohort samples. In the UK Biobank cohort sample, higher educational levels were associated with lower odds of SSRI switching. In the total sample, severe depression was nominally associated with SSRI switching status. Higher polygenic scores for antidepressant non-remission was associated with an increased risk of SSRI switching. Overall results show switching can be a proxy measure for nonresponse to SSRIs [Limitations in data availability and sample sizes in the UK Biobank primary care records. UK Biobank genetic analyses were underpowered, therefore larger sample sizes are needed to replicate findings. UK Biobank EHR data lack response measures available in clinical trials (i.e., depression symptom scores at baseline and during treatment). Potential for residual confounding since choice of treatment and choice to switch treatment have multiple contributing factors that were accounted for].	Patients with depression were identified using primary care diagnosis records for depressive disorders, using a previously validated algorithm (see PMID 33753889).
Zang et al. (2024) (22)	Compared the local performance and transportability of different ML-based suicide prediction models for children and adolescents, collected at different clinical touchpoints (inpatient, outpatient, and all encounters) [Claims data and EHR data from the APCD in Connecticut (*n* = 155,486), HIDD in Connecticut (*n* = 28,934), and KHIN (*n* = 100,900)].	Local performance: Regularized LR (simpler model) for suicide prediction was sufficient across APCD, HIDD, and KHIN datasets when trained and tested on the same data source. More complex ML models, LSTM especially, did not outperform regularized LR. Transport performance: All transported models showed either inferior or comparable performance compared to local models. The LSTM model showed inferior transport performance compared to regularized LR. LSTM showed comparable transported performance to other models only in the case of APCD to HIDD (No investigation of model calibration was performed, which is important for transportability, thus warranting the need for future work).	Three ML models were used for predictive modeling [regularized LR (simple), GBM (complex), and deep LSTM (complex)]. Both local and transported performance were assessed for each ML-predictive model.
Miranda et al. (2024) (25)	Characterized PTSD by leveraging the knowledge of the RDoC framework using clinical notes to understand how various RDoC domains manifest in different patient populations. RDoC domains were: arousal regulatory, cognitive systems, negative valence systems, positive valence systems, sensorimotor systems, social processes. Males were compared to females and military veterans were compared to non-veterans [Unstructured electronic medical record data (5.67 million clinical notes from 38,807 PTSD patients, University of Pittsburgh Medical Center)].	Females showed a higher prevalence of RDoC information than males (*p* = 0.03552), even when considering veteran status (veteran vs. non-veteran). The highest levels of information for females overall were sensorimotor systems and social processes, whereas the highest levels of information for males overall were negative valence systems, arousal regulatory, positive valence systems, and cognitive systems. A consistent decrease in instances across all RDoC domains was observed in PTSD patients after psychotherapy (vs. before). Clinical notes were qualitatively assessed to explore RDoC's impact on PTSD, finding the following most prominent RDoC keywords: stress, abuse, alcohol, sleep, suicidal, anxiety, hallucination, and attention [Study focused mainly on five out of the six RDoC domains, which limits applicability of the sixth domain (sensorimotor System), and is contextually confined to PTSD].	A natural language processing workflow was used to analyze electronic medical record data and identify and extract RDoC using a pre-trained transformer-based natural language model (all-mpnet-base-v2).
Yu-Lefler et al. (2024) (29)	Investigated among 4–7 year-old children (1) the trajectory and overall association of DBD outcomes as a function of PMT treatment engagement; and (2) demographic, clinical, and service risk factors associated with DBD behavioral outcomes within this PMT program. Measured average change in DBD behavior problem level from initial to last appointment by total attendance [U.S.-based outpatient behavioral clinic data (*n* = 1,211)].	When evaluating the proportion of patients reaching optimal DBD behavioral outcomes and first-to-last appointment change in DBD behavior problem rating, patients achieved a >50% reduction in DBD behavioral symptomology by 10 appointments involving PMT treatment engagement. Maximum treatment effect of 70% reduction was achieved by 24–29 appointments. Treatment effect reduced (26%–17%) by >30 visits. Over half (54%) of patients reached optimal DBD behavioral outcomes through consistent attendance [Findings not generalizable to the broader population; specific to one private, outpatient PMT clinic within a single pediatric hospital system. The study assessed typically developing children and involved retrospective data that did not inform whether children might have regressed by their last appointment].	None.
Liman et al. (2024) (26)	Explored how psychiatric comorbidities impact treatment change and response in adult patients with ADHD. Specifically, examine (1) the association of psychiatric comorbidities and patient characteristics with treatment change and response, and (2) the association of treatment change with HRU [U.S. electronic health record data within a longitudinal behavioral health real-world database (*n* = 3,387)].	Top occurring psychiatric comorbidities at baseline were MDD (40.6%), anxiety disorder (32.5%), substance use disorder (SUD; 19.5%), and post-traumatic stress disorder (PTSD; 17.3%). Among Subset A (patients with at least 12 months of post-index visit data), 44.8% of patients experienced a treatment change within the 12-month follow-up period. Being prescribed both a stimulant and non-stimulant at index was significantly associated with increased risk of treatment change (*p* = 0.01). Interaction between age and index ADHD medication was found to be statistically significant (*p* = 0.007). Among Subset B (patients with at least 6 months of post-index visit data) an increase in HRU was associated with being prescribed non-stimulant (*p* = 0.01), having comorbid MDD (*p* = 0.001), or mood disorder (*p* = 0.004). A 10-year increase in age was associated with having a 4% decrease in HRU (*p* = 0.009) (Potential for unmeasured confounders and biases as well as inconsistent HRU due to the nature of RWD and unlike trial data. Non-pharmacological data (i.e., psychotherapy data) was not captured. Unable to capture patients who may have been prescribed ADHD and/or other relevant medications in the primary care setting. Use of composite utilization rates to gauge the overall HRU does not reflect real-world economic burden experienced by patients].	ADHD symptoms present within ±30 days from the index date were derived from mental state examination notes using natural language processing methods.
Pompili et al. (2023) (30)	Described clinical and demographic characteristics of patients with major depressive disorder and suicidality, treatment utilization pathways, and clinical and psychosocial outcomes over time. Also captured relevant HRU and the associated costs for a subgroup of patients [Administrative data retrieved from Italian Local Healthcare Unit databases and prospectively collected health records across health centers (*n* = 133, prospective analysis; *n* = 41, administrative patient data analysis; *n* = 18, caregiver analysis)].	Patients [mostly female (68.4%), mostly with middle to high school level education (75.9%), and mostly cohabiting with spouse/partner/parents/friends (82.7%)] described past personal life events (53.5%), marital separation/divorce (27.9%), or death of a close family member or a friend (24.6%). Most had no prior history of suicidality (67.7%) and recurrent depressive episode (71.6%). Most patients received antidepressants (78.8%) or antipsychotics (68.2%) as first and last observed (31 days following first) treatment indication. Only 16.7%–19.3% of patients received psychopharmacotherapy with psychosocial treatment during the first to last observed treatment indication. Among the 41 patients included in the substudy 92.7% had one or more psychiatry-related hospitalizations (16.4 ± 27.5 days’ stay) in the 3 years prior. Over half (54.9%) patients accessed emergency care at least once during the prospective observational period. Mean costs per patient were 2,112 € ± 1,461 € during the observational period (Study sites were not randomly selected. Patient evaluation was based solely on clinical judgment. Patient sample not generalizable to the Italian population).	None.
Sadeh-Sharvit et al. (2022) (27)	Explored how common clinical session summaries are in real-world behavioral treatments (17,607 behavioral treatment sessions in the United States).	Clinical sessions summaries present at only 0.3% (54 out of a total of 17,607 behavioral treatment sessions). Session summaries most commonly addressed interpersonal relationships with family and friends (*n* = 27), issues related with work (*n* = 20), the word “change” (*n* = 6), and alcohol (*n* = 5). Sessions with a summary had a higher listening ratio than those without (49% vs. 33%) (Low number of sessions with summary statements limited ability to perform sentiment and content analyses. Analysis did not include patient outcome data like symptom reduction or client satisfaction).	Clinical sessions were processed via an AI therapy-specific platform that generates a verbatim session transcript and summarizes intervention insights. ML and natural language processing used to collect key metrics from treatment sessions and integrate them with standardized evidence-based self-report measures.
Kern et al. (2021) (23)	Examined treatment patterns within the year following suicide attempt or suicidal ideation (1-year post-index period) among patients diagnosed with major depressive disorder [U.S.-based commercial administrative insurance claims data (*n* = 42,204)].	Most (84.4%) of patients were treated with at least one class-based regimen at any point during the 1-year post-index period. Among those treated, most (70.2%) received a subsequent class-based regimen, while 46.3% received a third and 28.1% received at least four. SSRIs were the most commonly received treatment class (any time during one-year post-index period, both first and second line of therapy; 61.9%) followed by bupropion and trazodone (51.3%), anxiolytics (50.8%) and anticonvulsants (43.6%) (Study focused solely on prescription medications rather than clinical procedures).	Validated algorithms with positive predictive values ranging between ranging from 70% to 100% were used to identify suicide attempts.

ADHD, attention-deficit/hyperactivity disorder; AI/ML, artificial intelligence or machine learning; APCD, all-payer claims database (connecticut); DBD, early childhood disruptive behavior disorders; GBM, gradient boosting machine; HRU, health resource utilization; HIDD, hospital inpatient discharge database (connecticut); KHIN, Kansas health information network; PMID, PubMed ID; PTSD, post-traumatic stress disorder; RF, random forest; RDoC, research domain criteria; LR, regularized logistic regression; LSTM, long-short-term memory neural network; PMT, parent management training; RWD, real-world data; SSRI, selective serotonin reuptake inhibitors; WESAD, wearable stress and affect detection.

One study characterized post-traumatic stress disorder (PTSD) using clinical notes and a framework to understand how various domains within that framework manifest differently across certain patient populations (25). Other studies focused on attention deficit hyperactivity disorder (ADHD) used RWD to estimate the long-term causal effect of ADHD medication initiation on national test scores among children (19), and explore how psychiatric comorbidities impact treatment change and response in adult patients with ADHD (26). Two studies centered on depression complexities, examining treatment patterns within the year following suicide attempt or suicidal ideation (one-year post-index period) among patients diagnosed with major depressive disorder (23) and developing a clinical phenotype characterized by prescription patterns in addition to clinical, demographic, and polygenic predictors of selective serotonin reuptake inhibitor switching (24).

In two studies, AI/ML was more front and center–one study assessed whether ML models for anxiety detection built using wearables data in controlled conditions could be applied to real-world environments (20) and another compared the local performance and transportability of different ML-based suicide prediction models for children and adolescents (22). Broadly across all nine studies, AI/ML applications comprised of: (1) algorithms used to estimate daily dosages and treatment duration, (2) traditional ML (including kernel *k*-means approach) and transfer learning models, (3) validated algorithms used to identify patients meeting study inclusion/exclusion criteria, (4) ML-predictive modeling with a focus on measuring local and transported performance, (5) natural language processing to analyze electronic medical record data and identify and extract data for qualitative assessment [including but not limited to patient symptoms], and (6) AI to transcribe and process notes from clinical sessions (19–27).

Studies without AI/ML components (3 of 12; 25%) involved EHR or clinical data (28, 29), and administrative data (30). Broadly, they evaluated RWD to explore: (1) disease trajectories; (2) behavioral or psychosocial outcomes in the presence or absence of psychiatry or other mental health therapeutic intervention(s); (3) demographic, clinical, and service risk factors associated with mental health-related outcomes; and (4) health resource utilization and associated costs among patient subgroups (28–30). Several aims were also reported across these studies utilizing RWD. One study collated lessons learned from a broader program assessing the effectiveness and implementation of a lifestyle-focused intervention for inpatients with mental illness (28). Another investigated real-world factors associated with behavioral outcomes amid parent management training treatment engagement and among children with early childhood disruptive behavior disorders (29). Lastly, one study leveraged RWD to describe clinical and demographic characteristics of patients with major depressive disorder; assess suicidality, treatment utilization pathways, and clinical and psychosocial outcomes over time; and capture relevant health resource utilization and associated costs across patient subgroups (30).

## Knowledge gaps around real-world complexity in mental health

The studies discussed herein are largely based in the United States or Europe, which may be due to the fact that North America and Europe have a high penetration of EHRs, integrated health systems, and national registries. In addition, these regions have available administrative/claims databases enabling real-world data studies. Studies from other regions should not only be anticipated and encouraged; they should be structurally supported through health data infrastructure development, expert consultation access, trustworthy community engagement, and peer-reviewed literature dissemination guidance and support.

Future work aiming to address gaps in our knowledge or understanding around the complexities of mental health care and services research using RWD should involve a combination of prospective RWE studies (including pre-registered study protocols) and robust systematic reviews and meta-analyses that account for robust RWD terminologies (e.g., EHRs, claims databases, patient-generated health data, pharmacy claims data, labs data, registry data). Critical appraisals or specific processes for obtaining and confirming data presented among study investigators are encouraged as well to ensure methodological rigor and study quality assurance. Below, we discuss in more detail actions and steps that can be taken toward (1) enhancing mental health research validity and reproducibility, and (2) facilitating transparency in RWE mental health services research.

## Discussion

### Action toward enhancing mental health research validity and reproducibility

Looking forward, we recommend two key actions to enhance mental health services research validity and reproducibility using RWD. First, researchers should consider relevant, reliable, and routine mental health RWD collection, curation, transformation, and analysis approaches and protocols. As demonstrated in Perez et al. (19), the target trial emulation (TTE) framework is an important methodological approach for RWD, as TTE utilizes observational data while emulating the structure of an RCT through a counterfactual framework for causal inference (11). By explicitly defining eligibility criteria, treatment strategies, follow-up periods, and outcomes, as if designing an RCT, a TTE framework allows researchers to apply principles and rules of trials when conducting an actual RCT is not feasible due to ethical, logistic, or financial constraints. The TTE framework is particularly important for mental health, where complex treatment pathways, comorbidities, and variable treatment adherence might obscure causal relationships.

Second, researchers should identify, create, or improve mental health-relevant data systems, including data system interoperability features, to capture the full range and scope of mental health care. For example, van Schothorst et al. recommend the following actions based on their learnings: standardizing and harmonizing data entry procedures; aligning data collection with routine care pathways; and using automated checks for real-time monitoring and correction to ensure data completeness. Improving mental health-relevant data systems using standardization and harmonization techniques for the purpose of aligning data collection with routine yet complex care pathways is, therefore, a critical action or step moving forward (28). Other critical steps must also involve the use of trustworthy, validated AI/ML techniques that may increase the speed, accuracy, and efficiency of mental health services research and care while enhancing value for patients and caregivers.

### Facilitating transparency in RWE mental health services research

To aid in setting up and communicating robust protocols for RWE studies, researchers should generally document their key study parameters using protocol standardization tools like the HARmonized Protocol Template to Enhance Reproducibility (HARPER) (31). Detailed protocols help clarify how mental health conditions are defined, how exposures (e.g., treatments, service use) and outcomes (e.g., symptom change, hospitalization, suicide attempts) are operationalized, and how biases specific to mental health care data—such as underdiagnosis, stigma-related under recording, or differential follow-up—will be addressed. When assessing whether RWD are suitable for mental health services research, tools such as the Authentic Transparent Relevant Accurate Track-Record (ATRAcTR) tool can be used to evaluate data quality (32).

Finally, when reporting analyses of observational data aimed at estimating causal effects, researchers should follow published guidance, including the Transparent Reporting of Observational Studies Emulating a Target Trial (TARGET) framework, which promotes clarity about the hypothesized intervention, timing of exposures and outcomes, and strategies for handling confounding, missing data, and censoring (33). RWE study registration on trustworthy public platforms like the RWE Registry (https://osf.io/registries/rwe) and, where applicable, clinicaltrials.gov is also recommended to ensure study protocols and other study design elements and plans are open and transparent.

## Conclusion

Real-world data offer a critical yet underutilized opportunity to address the growing global burden of mental illness by capturing the complexity of mental health care as it is delivered. Here, we highlight a few RWD-driven studies on complex mental health, all of which are concentrated in the United States and Europe, methodologically heterogeneous, and uneven in the use of AI/ML. Future research should expand RWD-based mental health services studies to underrepresented regions, rigorously evaluate and refine AI/ML tools for real-world clinical use, and systematically compare advanced causal inference approaches—such as target trial emulation—across diverse data environments and care pathways. Additional priorities include developing and testing interoperable, standardized mental health data systems and examining how social, cultural, and structural determinants can be more completely captured and linked within RWD sources. Adoption of protocol and reporting frameworks such as HARPER, ATRAcTR, and TARGET, along with prospective registration of RWE studies, will be essential to improving validity, reproducibility, and credibility. Collectively, these actions and areas of future research can accelerate a more equitable, data-informed transformation of mental health services, ultimately supporting more timely, personalized, and effective care for people living with mental health conditions worldwide.
